# Self‐harm in individuals with substance use disorders: Predictive factors and risk model

**DOI:** 10.1111/add.70465

**Published:** 2026-05-07

**Authors:** Rongqin Yu, Derek K. Tracy, Julia Sinclair, Isabell Brikell, Paul Lichtenstein, Henrik Larsson, Seena Fazel

**Affiliations:** ^1^ Department of Psychiatry University of Oxford Oxford UK; ^2^ South London and Maudsley NHS foundation Trust London UK; ^3^ Department of Psychosis Studies Institute of Psychiatry, Psychology & Neuroscience, King's College London London UK; ^4^ Department of Psychiatry, Faculty of Medicine University of Southampton Southampton UK; ^5^ Department of Medical Epidemiology and Biostatistics Karolinska Institute Stockholm Sweden

**Keywords:** substance use disorders, self‐harm, risk factors, risk prediction, national registers, cohort studies

## Abstract

**Background and aims:**

Substance use disorders are associated with an elevated risk of self‐harm. Currently, clinical and structured assessment of self‐harm risk typically relies on evidence from the general population samples. The aim of this study was to develop a risk model for self‐harm that incorporates predictors specific to individuals with substance use disorders.

**Methods:**

Using national registers, we identified a population‐based cohort of 449 720 individuals with substance use disorders in Sweden between 2006 and 2020. We tested independence and strength of a range of socio‐demographic and clinical factors, obtained through linkage of population‐based registers, with a Cox proportional hazards model, and estimated the risk of self‐harm. For the risk model, 361 120 individuals were allocated to the development sample and 88 600 to external validation based on different geographical regions. We assessed self‐harm risk over five predetermined follow‐up periods—within 7 days, 1 month, 3 months, 6 months and 12 months—following a healthcare contact for substance use disorders.

**Results:**

In the development sample, self‐harm rates ranged from 0.6% to 3.5%, and in the validation sample from 0.5% to 3.6%. Ten risk factors were retained in the final risk model. Strongest associations with subsequent self‐harm were for clinical factors: previous self‐harm [hazard ratio (HR) = 3.17, 95% confidence interval (CI) = 3.08–3.26] and comorbidity of mental disorders (HR = 2.63, 95% CI = 2.50–2.72). Recent psychotropic medication use, including antidepressant (HR = 1.29, 95% CI = 1.23–1.38) and antipsychotic treatments (HR = 1.34, 95% CI = 1.24–1.44), was associated with increased risk, even after adjusting for psychiatric comorbidity, likely reflecting greater clinical severity and complexity. Across follow‐up periods, performance was good in terms of discrimination, with area under the curve (AUCs) ranging from 0.73 (95% CI = 0.71–0.76) to 0.79 (95% CI = 0.78–0.80). In relation to calibration, expected‐to‐observed risk ratios were 1.00 to 1.04 and Brier scores 0.01 to 0.04 across follow‐up periods. We used the model to generate a simple web‐based risk calculator [Oxford Self‐hArM after substance use disorders (OxSAMS)].

**Conclusions:**

Modifiable clinical factors appear to have the strongest associations with increased risk of self‐harm in people with substance use disorders. Structured tools, taking account of the different strengths of those factors, could inform clinical decision‐making and provide a baseline assessment for training and research

## INTRODUCTION

Substance use disorders (SUDs) are associated with more than a 10‐fold increased risk of self‐harm compared with general population samples [1] and represent a significant and potentially preventable cause of morbidity. Accurate assessment of this risk can potentially identify modifiable factors and individuals who would benefit from early intervention.In addition, care pathways and resource allocation can be improved with better risk identification, which are key challenges in healthcare systems with competing demands on limited funding for these services.

Although risk assessment tools for self‐harm and suicide exist [2, 3], none are tailored specifically to individuals with SUDs. A range of relevant risk factors have been identified, including socio‐demographic characteristics, clinical history (e.g. recent treatment for substance use) and psychiatric comorbidities [4, 5, 6]. However, existing research has been limited by small sample sizes and selective cohorts, such as hospitalised patients or military veterans [7]. The lack of large‐scale, population‐based data limits the identification of modifiable risk factors, information on their relative effect sizes and development of accurate risk assessment models. Currently, assessment of risk typically relies on unstructured and subjective clinical judgement, the accuracy and impact of which is uncertain [8, 9]. There is a need for risk models tailored to people with SUDs, developed using sufficiently large, representative samples [10].

This study aims to address this gap by developing a risk assessment model using a large, nationally representative sample of individuals with SUDs, applying high‐quality, evidence‐based methods. Such a tool could complement and augment clinical decision‐making by anchoring risk assessments in empirical data, improving consistency between and across services and supporting triage, clinical care and discharge planning. By accurately stratifying risk, such a model could also potentially improve resource allocation.

## METHODS

We identified a cohort of individuals with a diagnosis of substance use disorders [alcohol and drug use disorders, International Statistical Classification of Diseases (ICD) codes: F10–19] in the Swedish Patient Register [11]. We identified in total 449 720 individuals meeting these criteria between 1 January 2006 and 31 December 2020, the most recent date for which registry data were available. The study was approved by the Regional Ethics Committee at Karolinska Institutet (2020–06540), which waived the need for informed consent as anonymized register‐based data were used.

### Procedures

We followed individuals from the date of their initial secondary healthcare contact for SUD until the first occurrence of an emergency hospital or specialist care visit (psychiatric or otherwise) for self‐harm, death, emigration or the end of the follow‐up period (31 December 2020). For individuals with more than one recorded contact of SUD in our data, we randomly selected one event to serve as the index episode (and therefore, it was not necessarily the initial specialist/secondary healthcare contact). National registers were linked using unique personal identification numbers, allowing accurate linkage of information on risk factors for each individual [11].

### Outcomes

Outcomes were defined as self‐harm within a range of time periods, 1 week (7 days), 1 month, 3 months, 6 months and 12 months following the index episode date. Consistent with previous research [3], self‐harm was identified using ICD‐10 codes for both non‐fatal and fatal self‐harm (ICD‐10 codes for intentional self‐harm: X60–X84, and events of undetermined intent: Y10–Y33, excluding Y34 for unspecified events). The inclusion of undetermined intent codes is standard practice in self‐harm and suicide research to reduce potential misclassification of intent [12]. These codes were obtained from the National Patient Register and Cause of Death Register.

### Risk factors

We tested effects of potential risk factors identified from the literature examining associations between SUDs and self‐harm [3, 13], and from consultation with potential users (e.g. clinicians working in SUD services). Risk factors included socio‐demographic characteristics (e.g. age at time of SUD diagnosis), clinical history and treatment (e.g. antidepressant treatment), family psychiatric history, previous self‐harm (including method) and any history of criminal arrest before the index episode (see Table S1).

Sample size considerations included more than 10 outcomes per predictor for model development and more than 100 outcomes for validation [14, 15]. Blinding for outcomes and predictors was ensured by independently extracting information for each relevant variable. Data for both risk factors and outcomes were obtained through linkage of population‐based Swedish registers: Total Population Register, Multi‐Generation Register, Patient Register (healthcare episodes in secondary care in the public system), Cause of Death Register, Longitudinal Integrated Database for Health Insurance and Labour Market Studies, Prescribed Drug Register, National Crime Register and Register of Persons Suspected of Offences [11].

### Statistical methods

We conducted multi‐variable Cox proportional hazard regression to examine the association of socio‐demographic, clinical, treatment and history of self‐harm–related factors with self‐harm outcomes, while accounting for time to event. Follow‐up ended at date of first self‐harm event after the index episode or at end of cohort follow‐up (31 December 2020), whichever occurred first. The primary research question and analysis plan were written up in a study protocol, but not pre‐registered on a publicly available platform;, therefore, the results might be interpreted as exploratory (although external validation was concluded).

SUDs were modelled together because of the substantial comorbidity and overlap across substance categories. Polysubstance use and multiple SUD diagnoses are common [16], and care for SUD is often delivered through integrated services rather than substance‐specific pathways [17]. We separated the cohort into development and validation samples using a stratified random selection based on residential region at the year of SUD diagnosis [18]. Stratification ensured balanced representation of urban and rural areas, with approximately 20% of individuals assigned to the validation sample and the remainder to the development sample. The larger development sample was retained to optimize model estimation, reduce the risk of overfitting and ensure stable predictor estimates, while maintaining adequate power for performance evaluation in the validation sample. The validation sample was selected to ensure balanced representation of urban and rural regions and areas of varying population size, with the remaining cohort allocated to model development. We used the development sample to generate models for predicting self‐harm outcomes after diagnosis, and the validation sample to test predictive performance in an independent subset of individuals from the same national cohort who were not used during model derivation. This design enabled evaluation of model performance in a representative population distinct from the development sample.

To account for missing values, we conducted 20 imputations [19], with regression models that used data from other factors and the studied outcome, using the Nelson‐Aalen cumulative hazard function. Missingness was minimal (3% for low income and unemployment), as predictor variables were routinely collected in national registers by professional and administrative personnel [11]. Missing values were assumed to be missing at random, and estimates were obtained by combining results across the imputed datasets.

Multi‐variable Cox proportional hazards regression analyses were conducted in the development sample, including all candidate predictors described above. Variables that remained statistically significant (2‐sided *P* < 0.05) were retained in the final model to ensure that selection reflected both statistical robustness and clinical relevance. To evaluate the predictive performance of the final model, we assessed both discrimination and calibration in both the development and validation samples across the same pre‐specified follow‐up periods (7 days, 1 month, 3 months, 6 months and 12 months). Performance in the development sample provides an estimate of apparent model performance, whereas evaluation in the validation sample assesses performance in an independent subset of the cohort and examines generalisability. We used Harrell's C‐index as an overall measure of discrimination [20], defined as the model's ability to distinguish between individuals with and without self‐harm outcomes during follow‐up. The C‐index ranges from 0.5 to 1.0, with 1.0 representing perfect discrimination. We calculated the area under the curve (AUC) for outcomes and estimated absolute predicted probabilities based on the regression model coefficients and baseline survivor function during the follow‐up. We assessed calibration using calibration plots comparing predicted and observed risks. We also calculated the Brier score [21], defined as the mean squared difference between predicted probabilities and observed binary outcomes (range 0–1, with lower values indicating better calibration). We reported sensitivity, specificity, positive predictive value, negative predictive value and 95% CIs using pre‐specified thresholds (1%, 3%, 5% and 10%) for self‐harm outcomes.

We used the final models to develop an online risk calculator, Oxford Self‐Harm after Substance Use Disorders (OxSAMS), available at https://oxrisk.com/oxsams/. The calculator applies the regression coefficients and baseline survival function from the final Cox model to generate individual predicted probabilities. In the validation phase, we applied the regression coefficients and baseline survival function from the final model estimated in the development sample to the validation sample and assessed model performance. All analyses were conducted using STATA statistical software, version 18 (StataCorp).

## RESULTS

The cohort consisted of 449 720 individuals (mean age = 45.3 years, SD = 20.1), with 361 120 assigned to the model development sample and 88 600 to external validation. The distribution of substance use disorder diagnoses (ICD‐10 F10–F19) in the development and validation samples is presented in Table S2. In the development sample, alcohol use disorders (F10) were most common (54.5%), followed by tobacco use disorders (18.5%) and multiple/other psychoactive substance use disorders (9.7%). A similar distribution was observed in the validation sample, with alcohol use disorders accounting for 50.0%, tobacco use disorders 21.9% and multiple/other psychoactive substance use disorders 11.5% (Table S2). In development, 28 566 (7.9%) individuals self‐harmed during a mean follow up time of 6.2 years. In the validation sample (*n* = 88 600), the corresponding figure was 7239 (8.2%) within a mean follow up of 6.1 years. The median age was 45.3 years (SD = 20.1) in the development sample and 45.1 years (SD = 20.0) in external validation, with the proportion of females being 38.4% and 40.0%, respectively. Table 1 shows the baseline characteristics of the development and validation samples, including socio‐demographic, clinical, mental health and crime factors. The two samples had similar baseline characteristics and self‐harm rates across follow‐up periods (Table 1 and Table S3).

**TABLE 1:** Baseline characteristics of individuals diagnosed with substance use disorders.

Characteristics	Development sample (*n* = 361 120)		Validation sample (*n* = 88 600)	
Age in years (SD)	45.2	SD = 20.1	45.1	SD = 20.0
Gender (% female)	138 672	38.4%	35 427	40.0%
Unemployment (%)	209 710	60.0%	53 070	61.4%
Low income (%)	119 181	34.2%	28 499	33.1%
Comorbidity of mental disorders	174 246	48.3%	44 516	50.2%
Antidepressant treatment (6 months)	14 286	4.0%	3459	3.9%
Antipsychotic treatment (6 months)	4580	1.3%	996	1.1%
Other psychotropic treatment (6 months)	37 610	10.4%	8401	9.5%
Previous self‐harm	41 499	11.5%	11 584	13.1%
Cutting as a self‐harm method	4224	1.2%	1108	1.3%
Arrest in the last 10 years	134 491	37.2%	31 773	35.9%

*Note*: SD, standard deviation.

**FIGURE 1:**
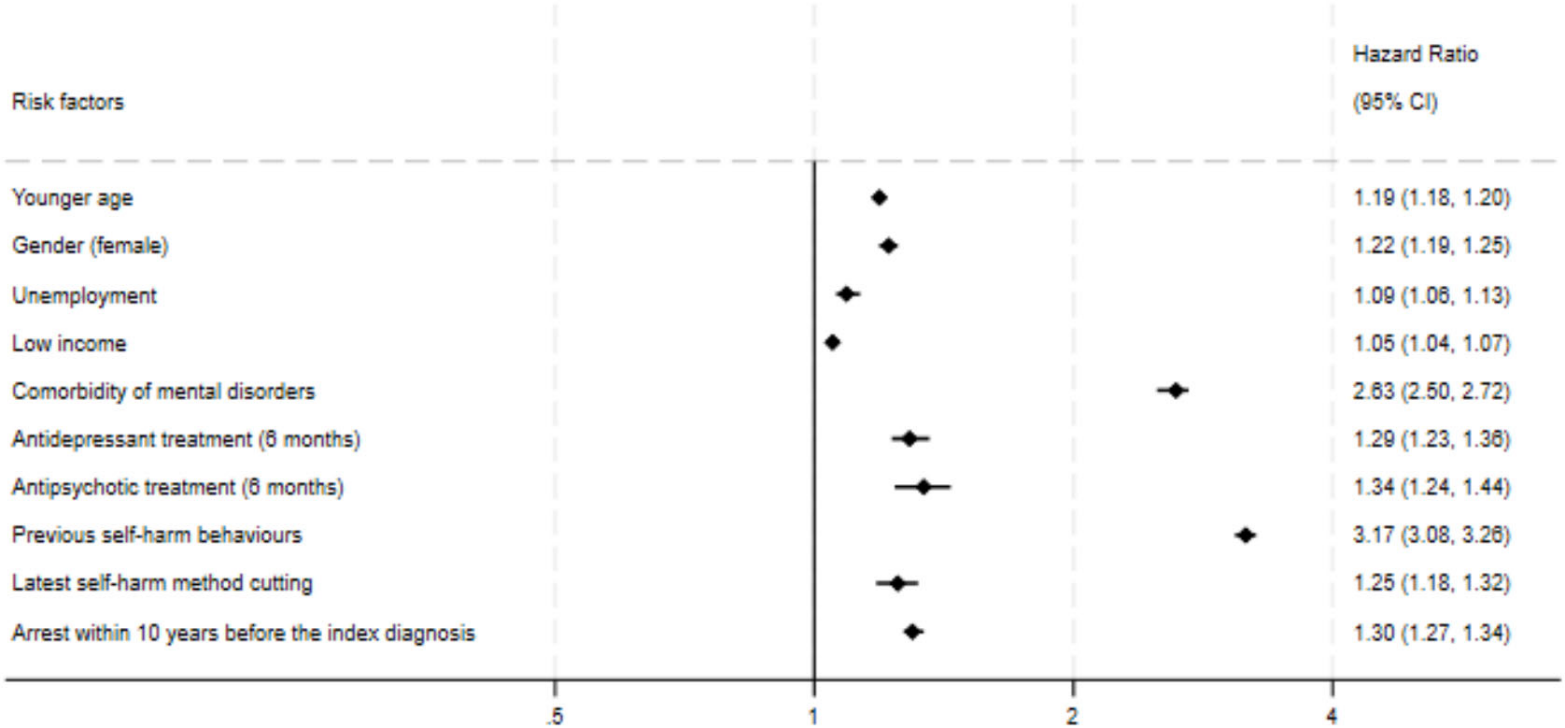
Risk factors and hazard ratios for self‐harm in substance use disorders. Note: Based on multi‐variable Cox regression model for the whole sample.

We tested a range of potential risk factors and associations with subsequent self‐harm after SUD healthcare contact. Adjusted hazard ratios (aHRs) ranged from 1.05 for low income to 3.17 for previous self‐harm behaviours. The strongest risk factors were previous self‐harm behaviours (aHR = 3.17; 95% CI = 3.08–3.26) and comorbidity of mental disorders (aHR = 2.83; 95% CI = 2.50–2.72). The final multi‐variable risk model for self‐harm included: age, sex (female versus male), unemployment, income, comorbidity of mental disorders, antidepressant treatment (in the 6 months before the index SUD episode), antipsychotic treatment (in the 6 months before the index SUD episode), previous self‐harm behaviours, self‐harm method involving cutting, and arrest within 10 years of the index SUD diagnosis. Figure 1 shows the hazard ratios (HRs), and see Table S4 for the model coefficients. Additional candidate predictors not retained in the final model showed limited or modest associations with self‐harm. Marital status (HR = 1.00; 95% CI = 0.84–1.19) and antipsychotic treatment at 3 months (HR = 1.00; 95% CI = 0.98–1.06) were not significantly associated. Antidepressant treatment showed a modest association (HR = 1.19; 95% CI = 1.01–1.35) but overlapped with the corresponding 6‐month measure. Parental self‐harm also showed a modest association (HR = 1.20; 95% CI = 1.10–1.20), but was not retained because of limited data availability when used clinicially and no additional improvement in model performance.

**FIGURE 2:**
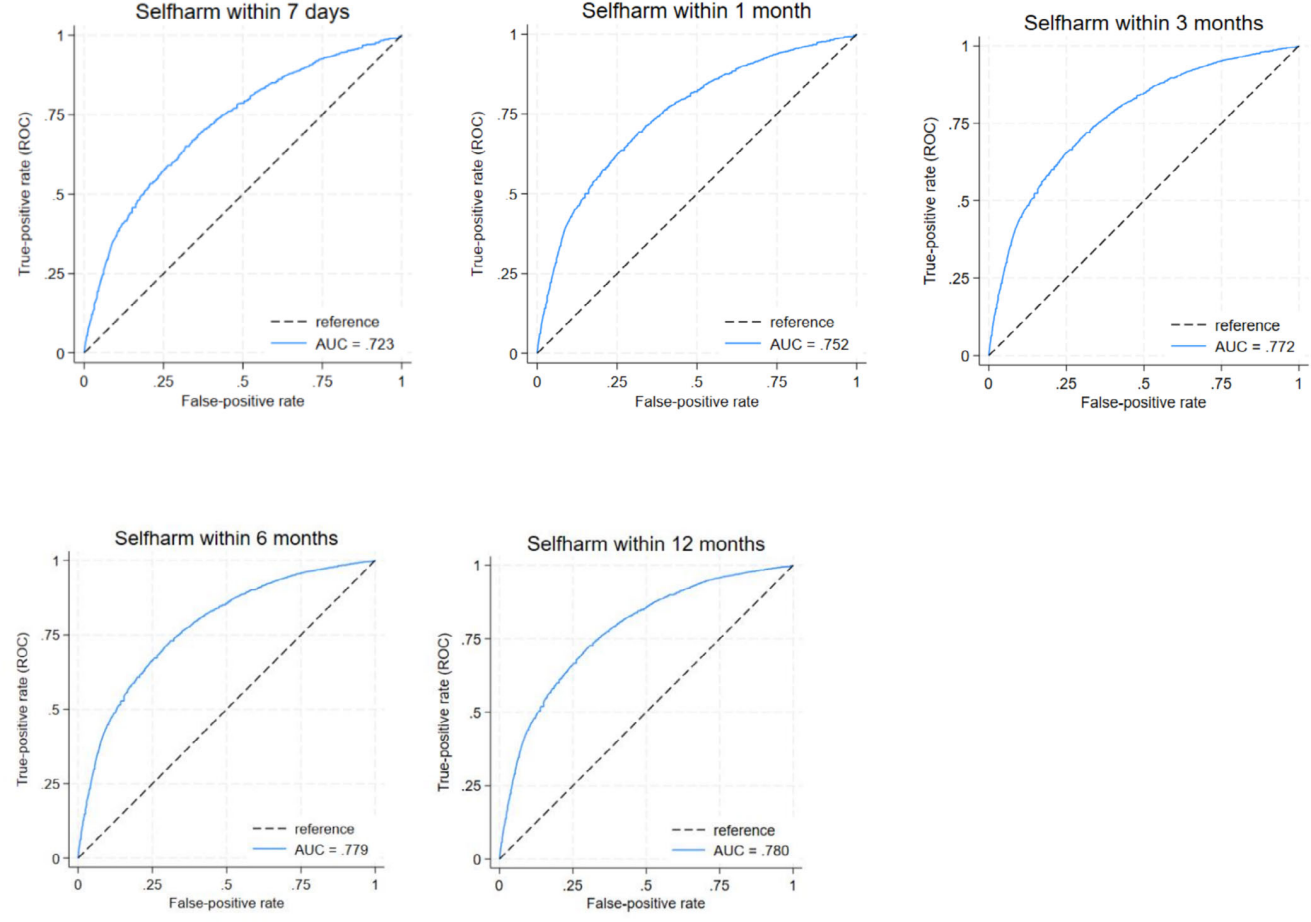
Model discrimination in predicting self‐harm among individuals with substance use disorders in the development sample.

The model demonstrated good discrimination performance in the developmental sample. The overall C‐index was 0.76 (0.75–0.77), indicating good overall discrimination. The derivation model showed good discrimination across pre‐specified time periods—7 days, 1 month, 3 months, 6 months, 12 months—with C‐indices ranging from 0.72 (95% CI = 0.71–0.73) to 0.78 (95% CI = 0.77–0.78) (Figure 2). Sensitivity analyses restricting the outcome to intentional self‐harm showed AUCs of 0.74 (95% CI = 0.71–0.76) for risk within 7 days, 0.78 (95% CI = 0.77–0.80) for risk within 1 month, 0.81 (95% CI = 0.80–0.82) for risk within 6 months and 0.81 (95% CI = 0.80–0.82) for risk within 12 months. Other discrimination measures, including sensitivity, specificity, positive predictive value and negative predictive value for pre‐specified cut‐offs (1%, 3%, 5% and 10%) for self‐harm within 12 months, are presented in Table 2. Lower cut‐offs were associated with higher sensitivity, but lower specificity (e.g. at 1%: sensitivity = 97.3%, specificity = 17.6%), whereas higher thresholds improved specificity at the expense of sensitivity (e.g. at 10%: specificity = 92.9%, sensitivity = 37.1%). A pragmatic balance in our data was approximately the 3% and 5% cut‐offs: at 3%, sensitivity = 77.4% and specificity = 63.5%; at 5%, sensitivity = 60.1% and specificity = 80.5%.

**TABLE 2:** Performance of the risk score in predicting 12‐month self‐harm among individuals with substance use disorders.

Self‐harm within 12 months					
Cut‐off scores	Sensitivity (95% CI)	Specificity (95% CI)	PPV (95% CI)	NPV (95% CI)	Accuracy (95% CI)
1%	97.3% (97.0–97.6)	17.6% (17.5–17.7)	4.3% (4.3–4.3)	99.4% (99.4–99.5)	20.5% (20.4–20.6)
3%	77.4% (76.6–78.1)	63.5% (63.3–63.6)	7.4% (7.4–7.5)	98.7% (98.6–98.7)	64.0% (63.8–64.1)
5%	60.1% (59.2–60.9)	80.5% (80.4–80.6)	10.5% (10.3–10.6)	98.2% (98.1–98.2)	79.8% (79.6–79.9)
10%	37.1% (36.3–37.9)	92.9% (92.8–93.0)	16.6% (16.2–16.9)	97.5% (97.5–97.5)	90.9% (90.8–91.0)

*Note*: 95% CI, 95% confidence interval; NPV, negative predictive value; PPV, positive predictive value.

**FIGURE 3:**
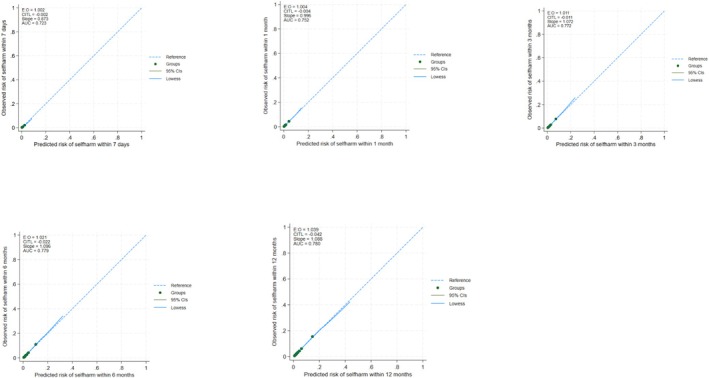
Model calibration for predicting self‐harm after substance use disorders in the derivation sample.

There was evidence of good calibration for the risk model (Figure 3). The expected‐to‐observed risk ratios ranged from 1.00 to 1.04 across different time periods (i.e. from 7 days to 12 months), and the Brier scores ranged from 0.01 to 0.04 (with a Brier score of 0 indicating perfect calibration). All Brier scores were lower than those obtained using either the mean predicted probability or a fixed value of zero (Table 3). External validation also showed good discrimination for self‐harm across different time periods (Figure S1), with AUCs ranging from 0.73 (95% CI = 0.71–0.76) to 0.79 (95% CI = 0.78–0.80), and good calibration (Figure S2). In addition, sensitivity analyses examining discrimination across broad SUD categories for risk within 12 months showed that performance was highest in alcohol‐related disorders (AUC = 0.77; 95% CI = 0.76–0.79), similar in tobacco‐related disorders (AUC = 0.76; 95% CI = 0.71–0.81) and more modest in other drug use disorders combined (AUC = 0.71; 95% CI = 0.69–0.72).

**TABLE 3:** Calibration performance for self‐harm prediction in individuals with substance use disorders at different follow‐up periods.

Time period (within)	7 days	1 month	3 months	6 months	12 months
Brier scores					
Predicted probability	0.0059513	0.0104252	0.0172683	0.0239485	0.0331453
Mean probability	0.0058006	0.0102550	0.0172328	0.0242163	0.0340412
Zero probability	0.0058346	0.0103622	0.0175399	0.0248311	0.0352791
E/O risk ratio	1.002	1.011	1.011	1.021	1.039

Abbreviation: E/O risk ratio, expected‐to‐observed risk ratio, used to assess calibration of the prediction model.

## DISCUSSION

Using population‐based data from Sweden, we investigated risk factors for self‐harm in 449 720 individuals diagnosed with SUDs. In addition, we developed and externally validated a risk model. Approximately 8% (27 571 individuals) self‐harmed over the average follow‐up of 6 years. The final risk model, based on 10 factors, had good performance (as indicated with a C‐index of 0.76 and calibration measures).

Clinical factors were more strongly associated with self‐harm risk than demographic ones, indicating their central role in identifying individuals at elevated risk. The strongest clinical predictors included previous self‐harm behaviours (aHR = 3.17), comorbidity of mental disorders (HR = 2.63) and recent antipsychotic (HR = 1.34) or antidepressant treatment (HR = 1.29). The associations with recent pharmacological treatment warrant careful interpretation. These findings do not suggest a causal relationship. Rather, they reflect clinical recognition of heightened severity or risk, whereby individuals presenting with more complex symptoms—such as comorbid affective or psychotic features—are more likely to receive medication. This interpretation is consistent with our finding that comorbidity of mental disorders also emerged as a strong independent risk factor. In this context, medication use likely functions as a marker of elevated clinical concern, rather than a direct contributor to risk. We emphasize that these findings should not be misinterpreted as evidence against pharmacological intervention, which remains underused in some high‐risk populations. Instead, they highlight the complexity of interpreting associations in observational data, particularly in the presence of confounding by indication. Future research should aim to disentangle treatment effects from illness severity using designs that more robustly account for clinical decision‐making and treatment pathways.

Additionally, a history of arrest was associated with an elevated risk of self‐harm (HR = 1.30). This association may reflect underlying psychosocial instability, behavioural dysregulation or greater illness severity. Although justice involvement—including arrest history—has been shown to be a risk factor for self‐harm in custody [22], its routine assessment in clinical practice remains inconsistent, and it is not systematically incorporated into most structured risk assessment tools for clinical populations. This suggests a need to broaden the scope of risk assessments in clinical settings to include socio‐legal indicators alongside traditional clinical markers.

In contrast, demographic characteristics such as younger age (HR = 1.19), female gender (HR = 1.22) and low income (HR = 1.05) were associated with more modest increases in risk. These findings underscore the value of incorporating clinical indicators into risk models and assessment, and suggest that demographic variables alone may be insufficient for accurate risk stratification.

These results underscore the complex and multi‐factorial nature of self‐harm risk, reinforcing the value of integrated approaches to identification and prevention. However, they also emphasise the importance of good clinical assessment and the potential for interventions to modify future risk. The current risk model is the first to specifically assess risk of self‐harm in individuals with SUD. It is different from the Alcohol Use Disorders Identification Test (AUDIT) [23], which uses a simple additive scoring system to screen for current risk of hazardous drinking, rather than future risk, assigning equal weight to all items regardless of their relative importance. In contrast, our tool directly assesses self‐harm risk by statistically testing and weighting predictors based on thier estimated effects, and is a prognostic tool.

Our study builds on previous work in other high‐risk populations by developing specialized models and tools for individuals with SUD—a high‐risk group under‐represented in risk modelling research. For example, the OxSET [3] and OxSATS [2] models were developed to model risk of repeat self‐harm and suicide, respectively, following hospital‐presenting self‐harm. In addition, models such as OxMIS and OxMIV [24, 25] have been designed to assess suicide and violence risk in individuals with schizophrenia‐spectrum and bipolar disorders, further demonstrating the value of diagnosis‐specific approaches.

The current risk model extends and advances this line of work by focusing specifically on individuals with SUD, offering a more targeted and clinically relevant approach for a population with distinct patterns of risk. Effect sizes differed between this SUD cohort and a previous OxSET one (which comprised of individuals presenting to hospital with self‐harm). For example, prior self‐harm showed a markedly larger effect in our cohort (aHR = 3.2) than in OxSET, where lifetime history before the index episode was weaker (HR = 1.2). These differences reflect specific population characteristics and baseline risk profiles, and highlight the importance of developing and validating models within the specific populations that they are intended to be used in. Effect sizes differed between our SUD cohort and OxSET (individuals presenting to hospital with self‐harm).

Research has highlighted challenges in generalizability and practical utility for predictive models in mental health [26]. By using a comprehensive dataset and focusing on a specific high‐risk population, our study addresses many of these limitations and offers more reliable and clinically applicable tools. Furthermore, our approach of statistically testing and weighting predictors enhances transparency and interpretability—both of which are essential for clinical decision‐making.

### Clinical implications

We have reported classification measures of our tool for predicting self‐harm in individuals with SUDs, including sensitivity, specificity, negative predictive value and positive predictive value. The tool exhibited varying performance across different cut‐off scores. The use of different cut‐off scores can allow for clinicians to tailor thresholds according to resource availability and the specific goals of self‐harm risk assessment within this population. This may be useful in high‐demand clinical settings—such as emergency departments or substance use treatment facilities—where timely and accurate risk stratification is critical for effective intervention and care planning. Integration into electronic health record (EHR) systems could facilitate use, enabling real‐time risk assessment and personalized intervention planning. Emerging linked clinical informatics systems pooling individual EHR data to larger organisational health population data‐sets offer even greater potential gains through understanding localised patient ‘footprints’ and epidemiological patterns [27]. However, ensuring that clinicians are adequately trained to interpret the tool's outputs is critical to maximizing clinical utility.

Lower risk thresholds were associated with higher sensitivity and lower specificity, whereas higher thresholds improved specificity at the expense of sensitivity. This trade‐off underscores the relevance of selecting thresholds based on clinical context. In practice, the choice of threshold should be guided by whether calibrated probability scores are preferred to hard classifications and the service‐level consequences of true/false positives and negatives (capacity, pathways and intervention costs).

In terms of balancing false positives and false negatives, rather than assuming that false negatives necessarily carry greater harm, it may be more appropriate to pose this as a service‐specific question: in this setting, which error type leads to more adverse consequences? In many SUD services, false positives may be less problematic if the resulting responses are supportive (e.g. safety planning, proactive follow‐up) and if individuals can derive additional benefits from engagement (e.g. reduced accidents or other harms), making a slightly more sensitive operating point (e.g. 3%) acceptable. Clear communication between clinicians and patients that outputs are probabilistic can help align actions to risk and maintain therapeutic engagement. Probabilistic information is an alternative increasingly recommended as it captures most information and inherently includes the error rate in risk estimates. For example, a predicted 12‐month risk of 5% means that approximately 1 in 20 people with a similar profile will self‐harm in the next year, and 19 in 20 will not.

Furthermore, given the high prevalence of co‐occurring mental health conditions among individuals with SUDs [28, 29, 30], the model could assist in identifying self‐harm risk within these clinically complex subgroups and the importance of addressing co‐morbidities. In this sense, the model acts as a structured way of identifying current risks and should not be viewed solely as a prediction tool.

### Strengths and limitations

Strengths include using data from a large national cohort of individuals diagnosed with substance use disorders, spanning 15 years, allowing more consistent and reliable model performance compared to studies based on smaller, selected samples [31]. In addition, large sample size provided sufficient statistical power to model risk over short‐term (7 days, 1 month and 3 months) and longer‐term (6 and 12 months) follow‐up. Varying prediction horizons provide important information for use in different clinical settings with varying preventive priorities [32]. Furthermore, use of ICD codes to identify alcohol and drug use disorders enhances model generalisability.

Several limitations should also be noted. First, we were unable to examine the impact of certain factors associated with self‐harm, such as childhood maltreatment and the quality of family relationships [33], because of lack of reliable data in Swedish registries. This limitation is common in register‐based studies. Second, although more detailed, time‐varying information on treatment and service use may improve prediction in some settings [34], we sought to balance predictive accuracy with feasibility for implementation, as such data are usually not consistently available or readily operationalised at the point of care across services. We, therefore, focused on routinely recorded predictors, and future research should examine whether time‐varying measures provide incremental predictive value. Third, further external validation is needed to assess whether the developed tool can be applied in new settings, particularly in countries that differ significantly from the Nordic countries in service provision and population demographics, including ethnic diversity, socio‐economic distribution and age structure.

## CONCLUSION

We developed a brief, accurate risk model for assessing self‐harm among individuals with substance use disorder using routinely collected data. The OxSAMS tool can support decision‐making in substance use and mental health services and potentially contribute to the prevention of self‐harm in this high‐risk population.

## AUTHOR CONTRIBUTIONS

**Rongqin Yu:** Conceptualization; investigation; methodology; validation; formal analysis; project administration; visualization; writing—review and editing; writing—original draft. **Derek K. Tracy:** Writing—review and editing; investigation. **Julia Sinclair:** Investigation; writing—review and editing. **Isabell Brikell:** Data curation. **Paul Lichtenstein:** Data curation; writing—review and editing. **Henrik Larsson:** Data curation; writing—review and editing. **Seena Fazel:** Conceptualization; investigation; writing—review and editing; funding acquisition; supervision.

## DECLARATION OF INTERESTS

None.

## Supporting information


**Table S1.** Data sources for risk factor.
**Table S2.** Distribution of ICD‐10 Substance Use Disorder Diagnoses (F10–F19) in the Development and Validation Samples.
**Table S3.** Rates of Self‐Harm Among Individuals with Substance Use Disorders in the Development and Validation Samples.
**Table S4.** Model Coefficients for Predicting Self‐Harm in Individuals with Substance Use Disorders.
**Figure S1.** Receiver Operating Characteristic Curves for Model Discrimination in Predicting Self‐Harm Among Individuals with Substance Use Disorders in the Validation Sample.
**Figure S2.** Model Calibration for Predicting Self‐Harm After Substance Use Disorders in the Validation Sample.

## Data Availability

The data that support the findings of this study are available from Statistics Sweden. Restrictions apply to the availability of these data, which were used under license for this study. Data are available from the author(s) with the permission of Statistics Sweden.
