# Supplementary material for: Self‐harm in individuals with substance use disorders: Predictive factors and risk model
Source: Addiction. 2026 May 7;121(9):2424–32. doi: 10.1111/add.70465 (PMC13486929; doi:10.1111/add.70465)
Supplement: Supplementary file 1 [file ADD-121-2424-s001.docx]

**Table S1. Data sources for risk factors**

| Variable / Information | Register Source | Description |
| --- | --- | --- |
| Age | Total Population Register | Age at index substance use disorder (SUD) diagnosis |
| Sex | Total Population Register | Biological sex recorded at registration |
| Income | Longitudinal Integrated Database for Health Insurance and Labour Market Studies (LISA) | Annual individual disposable income |
| Unemployment | Longitudinal Integrated Database for Health Insurance and Labour Market Studies (LISA) | Employment status prior to index episode |
| Comorbidity of mental disorders | National Patient Register | Recorded psychiatric diagnoses prior to index episode |
| Antidepressant treatment (6 months prior) | Prescribed Drug Register | Dispensed antidepressant medication |
| Antipsychotic treatment (6 months prior) | Prescribed Drug Register | Dispensed antipsychotic medication |
| Previous self-harm | National Patient Register | History of self-harm diagnosis prior to index episode |
| Method of previous self-harm (cutting) | National Patient Register | ICD-10 coding specifying method |
| Arrest within 10 years prior to index episode | National Crime Register / Register of Persons Suspected of Offences | Criminal justice involvement prior to index episode |

**Table S2. Distribution of ICD-10 Substance Use Disorder Diagnoses (F10–F19) in the Development and Validation Samples**

| **ICD-10** | **Substance Category** | **Development (n=361,120)** | **%** | **Validation (n=88,600)** | **%** |
| --- | --- | --- | --- | --- | --- |
| F10 | Alcohol use disorders | 196,671 | 54.5 | 44,290 | 50.0 |
| F11 | Opioid use disorders | 16,626 | 4.6 | 4,070 | 4.6 |
| F12 | Cannabis use disorders | 18,418 | 5.1 | 3,224 | 3.6 |
| F13 | Sedative/hypnotic use disorders | 16,836 | 4.7 | 4,877 | 5.5 |
| F14 | Cocaine use disorders | 1,892 | 0.5 | 379 | 0.4 |
| F15 | Other stimulant use disorders | 6,959 | 1.9 | 1,625 | 1.8 |
| F16 | Hallucinogen use disorders | 1,311 | 0.4 | 373 | 0.4 |
| F17 | Tobacco use disorders | 66,873 | 18.5 | 19,420 | 21.9 |
| F18 | Volatile solvent use disorders | 432 | 0.1 | 132 | 0.1 |
| F19 | Multiple/other psychoactive substance use disorders | 35,102 | 9.7 | 10,210 | 11.5 |
| **Total** |  | **361,120** | **100.00** | **88,600** | **100.00** |

**Table S3. Rates of Self-Harm Among Individuals with Substance Use Disorders in the Development and Validation Samples**

| Time period | Development sample n=361,120 | Validation sample  n = 88,600 |
| --- | --- | --- |
| 7 days | 2107 (1800^a^) (0.6%) | 464 (413) (0.5%) |
| 1 month | 3742 (3207) (1.0%) | 915 (796) (1.0%) |
| 3 months | 6334 (4848) (1.8%) | 1536 (1131) (1.7%) |
| 6 months | 8967 (6729) (2.5%) | 2202 (1835) (2.5%) |
| 12 months | 12740 (9336) (3.5%) | 3215 (2550) (3.6%) |

*Note*: ^a^ Intentional self-harm

**Table S4. Model Coefficients for Predicting Self-Harm in Individuals with Substance Use Disorders**

| Risk factors | Coefficient | LCI | UCI |
| --- | --- | --- | --- |
| Younger age | 0.19 | 0.18 | 0.19 |
| Gender (female) | 0.12 | 0.10 | 0.15 |
| Unemployment | 0.11 | 0.08 | 0.13 |
| Low income | 0.08 | 0.06 | 0.09 |
| Comorbidity of mental disorders | 1.00 | 0.97 | 1.03 |
| Antidepressant treatment (6 months) | 0.30 | 0.22 | 0.37 |
| Antipsychotic treatment (6 months) | 0.29 | 0.22 | 0.36 |
| Previous self-harm behaviours | 1.19 | 1.16 | 1.22 |
| Latest self-harm method cutting | 0.22 | 0.16 | 0.28 |
| Arrest within 10 years before index diagnosis | 0.26 | 0.24 | 0.29 |

Note: LCI = Lower Confidence Interval; UCI = Upper Confidence Interval.

**Figure S1. Receiver Operating Characteristic Curves for Model Discrimination in Predicting Self-Harm Among Individuals with Substance Use Disorders in the Validation Sample**

**Figure S2. Model Calibration for Predicting Self-Harm After Substance Use Disorders in the Validation Sample**
